# Recreational Drone-Related Injuries in Children: A Review of National Electronic Injury Surveillance System (NEISS) Data

**DOI:** 10.7759/cureus.15390

**Published:** 2021-06-02

**Authors:** Abdullah Khan, Lance Brown

**Affiliations:** 1 Pediatric Emergency Medicine, Loma Linda University Medical Center, Loma Linda, USA; 2 Pediatric Emergency Medicine, Dignity Health - St. Rose Dominican Hospital, Siena Campus, Henderson, USA

**Keywords:** drones, children, injuries, neiss, recreational

## Abstract

Introduction

Drones are unmanned aerial vehicles controlled by a person on the ground, used for recreational purposes. The purpose of the study is to describe characteristics and patterns of injuries reported in children from recreational drones.

Methods

We extracted data from the National Electronic Injury Surveillance System involving (NEISS) over a period of 10 years from 2010 to 2019 regarding injuries to children for ages zero up to 18 years. We included the subjects with drone-related injuries. All other toy-related injuries were excluded. We applied descriptive statistics to calculate proportions and confidence intervals for categorical variables and median for continuous variables.

Results

We included a total of 26 subjects. In our sample, the number of male subjects (65%; n = 17) was higher than the number of females (35%; n = 9). Head and face were the commonly affected body parts (58%, n = 15). The most common diagnoses were lacerations (42%; n = 11) and contusions/abrasions (27%; n = 7). The majority of the subjects were treated and discharged from the emergency department (92%; n = 24). A significant number of injuries were caused by the direct impact of drones (65%; n = 17).

Conclusion

Drones have the potential to cause injuries. Precautions are warranted to decrease the incidence of these injuries in children.

## Introduction

Drones, also known as unmanned aerial vehicles (UAV) or unmanned aircraft systems (UAS), have become popular as recreational devices in recent years. There are more than one million registered recreational drones in the United States [1]. Drones are now a multi-billion dollars industry [2]. With the use of these drones, injuries are being reported [3,4]. Other than recreational activities, drones are also being tested for their utility in the field of emergency medicine. Their time to deliver automated external defibrillators is better than the traditional emergency medical services in out of hospital cardiac arrests [5,6]. They are also being considered for faster delivery of blood products and other emergency equipment [7,8]. Although drones are being considered for medical usage, injuries reported in the literature are from recreational drones. Our paper will also focus on recreational drones.

Drones are rapidly mobile vehicles with speeds up to 45 m/s and with mass up to 55 pounds (25 kilograms) as allowed by regulations [9]. With this speed and mass, the chances of impact injuries are high [10]. Skull fractures can occur from the direct impact of drones with speeds as low as 22 m/s [11]. The purpose of our study is to describe the pattern of injuries related to non-military recreational drones in children.

## Materials and methods

We conducted a retrospective review and descriptive analysis of data obtained from the National Electronic Injury Surveillance System (NEISS) in June 2020. NEISS is a database operated by the United States Consumer Product Safety Commission (CPSC), which tracks injuries reported from a representative sample of emergency departments across the country.

We searched NEISS using *toy vehicle* (excluding riding toys) with NEISS code 5021 for patients between zero and 18 years of age over a period of 10 years (2010 to 2019) [12]. Initially, we obtained 3,230 records in the form of Excel sheets for each year. We searched next for the words *drone*, *aircraft*, and *aerial vehicle* in the *narrative* section of each Excel sheet. With this search, 26 records were obtained and were included in the study. The remaining toy-related injuries were excluded.

For the subjects included, we retrieved the data on age, sex, race, diagnosis made in the emergency department, body parts affected, and disposition. The proportions are reported in percentages with a 95% confidence interval (CI), whereas continuous variables are reported in the median with the interquartile range (IQR). The mechanism of injuries was further categorized into two groups: *Injuries by the direct impact of drones* and i*njuries by other objects while handling drones*.

We used *PubMed, Medline and Google scholar *to search for drones-related injuries. We used the words *drones, aerial drones, unmanned drones* and combined these words with “*injuries*” and *children.* We were able to find three case reports on injuries related to children and two papers focused on the whole population but did not answer the questions regarding injuries related to drones in children [3,4,13-15]. We could not determine whether these case reports were reported to NEISS. So, we ran the analysis only on the NEISS data.

The research involved public data, and our Institutional Review Board (IRB) determined that this study did not meet the definition of human subject research, as the data obtained does not contain private individually identifiable information.

## Results

In our study, the age of subjects ranged from three months to 17 years. The median age is eight years (IQR 2.75-14). Out of the 26 subjects, 65% (n = 17) are male and 42% (n = 9) female. Regarding the race, 58% (n = 15) are white, and 11% (n = 3) are African American, whereas race is not recorded for 27% (n = 7) of the subjects (Table 1).

**Table 1:** Characteristics of subjects with drone-related injuries *One subject swallowed a battery, and the other subject inhaled smoke.

Categories	Sub-categories	Number of subjects (percentage with 95% confidence interval)
Sex	Male	17 (65, 42–88)
	Female	9 (35, 17–55)
Demographics	White	15 (58, 37–77)
	African American	3 (11, 2–30)
	Other	1 (4, 0–20)
	Not recorded	7 (27, 12–48)
Diagnosis	Laceration	11 (42, 23–63)
	Contusion/abrasion	7 (27, 12–48)
	Sprain	2 (8, 0–25)
	Blunt head trauma	1 (4, 0–20)
	Fracture	1 (4, 0–20)
	Ingested foreign object	1 (4, 0–20)
	Smoke inhalation	1 (4, 0–20)
	Unclear/not stated	2 (8, 0-25)
Body parts affected	Head and face	15 (58, 37–77)
	Upper extremity	4 (15, 4–35)
	Lower extremity	4 (15, 4–35)
	Trunk	1 (4, 0–20)
	Internal organs injuries*	2 (8, 0–25)
Disposition	Treated and discharged	24 (92, 75–99)
	Admitted	2 (8, 0–25)
Mechanism of injury	Injury by the direct impact of drones	17 (65, 44–83)
	Injury without direct impact of drones	9 (35, 17–56)

There were no drone injuries reported from 2010 to 2014. From 2015 to 2019, drone-related injuries were reported every year. Of all the drone-related injuries, one case (3%; 95 CI 0-20) was reported in 2015, four cases (15%; 95 CI 4-35) in 2016, six cases (23%; 95 CI 8-44) in 2017, nine cases (35%; 95 CI 17-56) in 2018, and six cases (23%; 95 CI 8-44) in 2019.

The most common diagnosis made in the emergency department was laceration, reported in 42% (n = 11) of subjects, followed by contusions and abrasions in 27% (n = 7). Sprains were reported in 8% (n = 2), whereas blunt head trauma and fracture were reported in 4% (n = 1) of subjects each (Table 1).

Among the body parts affected, the head and face were injured in 58% (n = 15) of subjects. Upper and lower extremity injuries were reported in 15% (n = 4) of subjects each (Table 1). There were two cases coded as* internal organ injuries*. One of these subjects, a nine-year-old female, accidentally swallowed a triple-A battery from a drone, and the other subject, a three-month-old baby, inhaled smoke when a drone battery exploded in the house (Table 2).

**Table 2:** Brief presentations of subjects with injuries sustained without direct impact by drones

Categories	Brief description
Injuries during retrieval of drone	Seven-year-old male threw a broom into the air that hit his face, causing facial laceration. He was trying to dislodge his drone from a tree.
	10-year-old female was climbing a tree to retrieve a drone and fell, causing left hip and lumbar pain.
	13-year-old male jumped off the roof to retrieve a drone from a tree, causing a tibial fracture.
	14-year-old male was climbing onto his roof to retrieve his drone when his hand slipped on a piece of metal, causing right-fifth-finger laceration.
Injuries while chasing drone	Eight-year-old was running while operating a drone in backyard and slipped, causing strain of knee.
	11-year-old male was chasing a drone in his yard and hit his knee on a metal ladder, causing knee laceration.
	14-year-old male stumbled while chasing a drone and inverted right ankle, causing ankle sprain.
Other	Nine-year-old female was holding a triple-A battery of the drone in her mouth and accidentally swallowed.
	Three-month-old female exposed to smoke from a battery explosion in a drone. Carboxyhemoglobin level 1.4.

Regarding disposition, 92% (n = 24) were treated and discharged from the emergency department, whereas two subjects were admitted to the hospital for further management. One of these subjects was 21 months old and was hit in the right eye by a drone, which caused the corneal laceration. The other subject was the nine-year-old female who accidentally swallowed a triple-A battery from a drone (Table 1). (This is the same case that was coded to have* internal organ injury*.)

We divided the mechanism of injuries into two broad categories. Out of 26 subjects in our study, 65% (n = 17) sustained injuries by* the direct impact of drones*, whereas 35% (n = 9) were injured* without the direct impact of drones* (Table 1). Among the latter, four subjects (44%; 95 CI 14-79) were injured while trying to retrieve a drone stuck on a tree or roof of a house, whereas three (33%; 95 CI 7-70) acquired injuries while chasing a drone (Table 2).

## Discussion

In our search, we found limited literature on injuries caused by the recreational uses of drones. Other than case reports, there were no articles describing patterns of injuries from drones in the paediatric population. In a case report by Chung et al., a 13-year-old male developed a skull fracture due to the impact of a drone [13]. In another case report, Moskowitz et al. described a nine-year-old with a ruptured right eye globe after being hit by a drone [14]. Similarly, Spitzer et al. reported cases of right-eye-globe rupture and conjunctival laceration in nine-year-old and 21-month-old, respectively, caused by drones [15]. All these cases reported injuries of head and face area by the direct impact of drones. Similarly, in our study, the head and face areas were the most affected body parts. But in a study looking at drone-related injuries in all ages, laceration to the hand and finger were noticed in 49% of their population, whereas in our study finger injuries were reported in only two cases [3].

Injuries have been reported to occur directly by impact of drones as well as indirectly while handling drones. According to Forrester, higher numbers of injuries are caused by direct impact [3,4]. Also, in our study, two-thirds of injuries were caused by the direct impact of drones. Johnson et al. reported that "Propeller injuries were the leading mechanism" for drone-related injuries and hobbyist aircraft injuries [4]. In another case report, an adult man was hit in the face by propellers of the drone and sustained extensive ocular injuries [16]. The percentage of hospitalization after drone-related injuries is at 2% according to previous studies [3,4]. In our study, 8% of the subjects were admitted to the hospital. This number can be a false representation considering the small number of subjects in our study.

The impact of different speeds and masses of drones has been studied on dummies and human cadavers [10,17]. Drones have the capacity to fly at five to six times faster speed than the speed required to cause skull fractures [17]. Previous literature discussed how increasing the mass of drones in an experimental environment increased the risk of concussion [10]. The drones with masses as low as 500 grams travelling at lower speeds can also produce enough energy to cause skull fractures [14]. Drones are regulated by the Federal Aviation Administration (FAA). As per regulations by the FAA drones can fly at altitude of 400 feet above the ground, with a maximum speed of 100 miles per hour [18]. At such speeds, and given the mass of the drones, chances of high-impact injuries increase.

Our study has several limitations. We have a small sample size. The data are derived from the NEISS database which provides a limited description of cases. There are chances of coding errors so the number of cases may be a misrepresentation of the actual incidences.

## Conclusions

In our study, we described characteristics of drone-related injuries in children to provide awareness in the era of the increasing use of drones. We conclude that lacerations and abrasions of the upper body, especially the head and face, are common drone injuries. A majority of drone-related injuries are easily treatable, but severe head and ocular injuries have been reported in the literature. These injuries are preventable. The FAA has elaborate guidelines for operating recreational drones. We suggest adhering to these guidelines. Further studies are needed to evaluate the utility of protective human equipment in operating recreational drones.

## References

[1] (2021). FAA Drone Registry Tops One Million. http://www.transportation.gov/briefing-room/faa-drone-registry-tops-one-million.

[2] (2021). The economic impact of unmanned aircraft systems integration in the United States. http://www.auvsi.org/our-impact/economic-report.

[3] Forrester MB (2019). Drone-related injuries treated at emergency departments. Am J Emerg Med.

[4] Johnson JA, Svach MR, Brown LH (2019). Drone and other hobbyist aircraft injuries seen in U.S. emergency departments, 2010-2017. Am J Prev Med.

[5] Claesson A, Fredman D, Svensson L (2016). Unmanned aerial vehicles (drones) in out-of-hospital-cardiac-arrest. Scand J Trauma Resusc Emerg Med.

[6] Pulver A, Wei R, Mann C (2016). Locating AED enabled medical drones to enhance cardiac arrest response times. Prehosp Emerg Care.

[7] Homier V, Brouard D, Nolan M (2021). Drone versus ground delivery of simulated blood products to an urban trauma center: the Montreal Medi-Drone pilot study. J Trauma Acute Care Surg.

[8] Mesar T, Lessig A, King DR (2018). Use of drone technology for delivery of medical supplies during prolonged field care. J Spec Oper Med.

[9] (2021). AC 107-2A - Small Unmanned Aircraft System (Small UAS) - Document Information. https://www.faa.gov/regulations_policies/advisory_circulars/index.cfm/go/document.information/documentID/1038977.

[10] Campolettano ET, Bland ML, Gellner RA (2017). Ranges of injury risk associated with impact from unmanned aircraft systems. Ann Biomed Eng.

[11] Stark DB, Willis AK, Eshelman Z, Kang YS, Ramachandra R, Bolte JH 4th, McCrink M (2019). Human response and injury resulting from head impacts with unmanned aircraft systems. Stapp Car Crash J.

[12] (2021). NEISS Coding Manual. https://www.cpsc.gov/s3fs-public/2019_NEISS_Coding_Manual.pdf.

[13] Chung LK, Cheung Y, Lagman C, Au Yong N, McBride DQ, Yang I (2017). Skull fracture with effacement of the superior sagittal sinus following drone impact: a case report. Childs Nerv Syst.

[14] Moskowitz EE, Siegel-Richman YM, Hertner G, Schroeppel T (2018). Aerial drone misadventure: a novel case of trauma resulting in ocular globe rupture. Am J Ophthalmol Case Rep.

[15] Spitzer N, Singh JK (2018). Pediatric ocular trauma caused by recreational drones: two case reports. J AAPOS.

[16] Crahay FX, Rampat R, Tonglet M, Rakic JM (2021). Drones' side effect: facial and ocular trauma caused by an aerial drone. BMJ Case Rep.

[17] Yoganandan N, Pintar FA, Sances A Jr, Walsh PR, Ewing CL, Thomas DJ, Snyder RG (1995). Biomechanics of skull fracture. J Neurotrauma.

[18] (2021). Recreational Flyers & Modeler Community-Based Organizations. https://www.faa.gov/uas/recreational_fliers/.

